# Outcomes of Atrial Fibrillation Ablation in Patients with Chronic Kidney Disease

**DOI:** 10.3390/jcm14176227

**Published:** 2025-09-03

**Authors:** Aharon (Ronnie) Abbo, Ziad Arow, Allon Eyal, Robert M. Glueck, Adi Elias, Roy Beinart, Eyal Nof, Feras Haskiah, Yoav Michowitz, Michael Glikson, Yuval Konstantino, Moti Haim, David Luria, Alexander Omelchenko, Keren Cohen-Hagai, Maysam Shehab, Ibrahim Marai, Avishag Laish-Farkash, Mahmoud Suleiman

**Affiliations:** 1 Cardiology Department, Rambam Health Care Campus, Haifa 3109601, Israelm_suleiman@rambam.health.gov.il (M.S.); 2 The Ruth and Bruce Rappaport Faculty of Medicine, Technion-Israel Institute of Technology, Haifa 3200003, Israel; 3 Cardiology Department, Meir Medical Center, Kfar Saba 4428163, Israel; ziad.arow@gmail.com (Z.A.);; 4 Heart Institute, Sheba Medical Center, Tel-Hashomer 5266202, Israel; 5 Faculty of Medicine & Health Sciences, Tel Aviv University, Tel Aviv 6997801, Israel; 6 Jesselson Integrated Heart Center, Shaare Zedek Medical Center, Jerusalem 9103102, Israel; 7 Faculty of Medicine, The Hebrew University, Jerusalem 9112102, Israel; 8 Cardiology Department, Soroka University Hospital, Beer Sheva 8410101, Israel; 9 Faculty of Health Sciences, Ben-Gurion University, Beer Sheva 8410501, Israel; 10 Cardiology Department, Hadassah Medical Organization, Jerusalem 9112001, Israel; 11 Nephrology Department, Meir Medical Center, Kfar Saba 4428163, Israel; 12 Department of Vascular Surgery, Meir Medical Center, Kfar Saba 4428163, Israel; 13 Cardiology Department, Baruch Padeh Medical Center, Tiberias 1528001, Israel; 14 The Azrieli Faculty of Medicine in the Galilee, Bar-Ilan University, Safed 1311502, Israel; 15 Cardiology Department, Assuta Ashdod University Medical Center, Ashdod 7747629, Israel

**Keywords:** atrial fibrillation, chronic kidney disease, pulmonary vein isolation, Israeli Catheter Ablation Registry (ICAR)

## Abstract

**Background:** Limited and inconsistent information exist about how kidney function affects the outcomes of ablation procedures in patients with atrial fibrillation (AF). Therefore, the aim of this study was to investigate the effectiveness and safety of AF ablation in a large national study across groups classified by varying levels of estimated glomerular filtration rates (eGFRs). **Methods:** The Israeli Catheter Ablation Registry (ICAR) is a prospective, multicenter cohort that includes patients who underwent pulmonary vein isolation (PVI) during the years 2019–2021 for the treatment of AF. Primary study endpoints were the recurrence of AF and the need for repeat ablation at 12 months. Secondary endpoints were rehospitalization and procedural complications after AF ablation. **Results:** Between January 2019 and December 2021, 1002 AF patients underwent PVI. Baseline creatinine was available in 929 patients, which comprised the study cohort. Of these patients, 226 (24%) had preserved eGFR (>90 mL/min/1.73 m^2^), 511 (55%) had mildly reduced eGFR (60–89 mL/min/1.73 m^2^), and 192 (21%) had moderately to severely reduced eGFR (<59 mL/min/1.73 m^2^). Patients with moderately to severely reduced eGFR were generally older and more likely to be female. There were no clinically meaningful differences in the use of antiarrhythmic medications among the eGFR groups, either before or after PVI. There were no significant differences in 12-month AF recurrence rates among the three study groups: 30%, 32%, and 40% in patients with preserved eGFR, mild, and moderately to severely reduced eGFR, respectively (*p* = 0.1). The one-year rehospitalization rate was higher in patients with moderately to severely reduced eGFR: 19%, 24%, and 32% in patients with preserved eGFR, mild, and moderately to severely reduced eGFR, respectively (*p* = 0.01). Periprocedural complications were infrequent across all the eGFR groups. Patients with an eGFR of <30 mL/min/1.73 m^2^ were underrepresented (<1%), limiting applicability to this group. **Conclusions:** PVI is a safe and effective procedure that should be considered for CKD patients with AF who are deemed as suitable for the intervention, even in the presence of declined eGFR values. Future studies are still needed to evaluate the safety and effectiveness of the procedure in individuals with severely reduced eGFR or end-stage kidney disease.

## 1. Introduction

Atrial fibrillation (AF) is the most common heart rhythm disorder, occurring more frequently in patients with chronic kidney disease (CKD) than in the general population [1,2]. AF and CKD often coexist, with a prevalence of 23.8% among CKD patients in the US and affecting half of those who also have heart failure (HF) [3]. Both conditions share risk factors and potential pathophysiological mechanisms, such as advanced age, male gender, obesity, smoking, physical inactivity, hypertension, ischemic heart disease (IHD), and HF [4,5,6,7]. The interplay between AF and CKD exacerbates cardiovascular complications and increases overall health risks: AF is associated with higher incidences of stroke, HF, and mortality, with these risks being even more pronounced in CKD patients who develop AF compared to the general population [8,9,10,11].

It has also been demonstrated that as the estimated glomerular filtration rate (eGFR) declines, the prevalence of AF increases [5,8]. Additionally, AF independently raises the risk of CKD [9,12], underscoring the need for vigilant monitoring and tailored management strategies in these patients.

According to several trials, catheter ablation is superior to antiarrhythmic therapy in preventing AF recurrence and improving survival [13,14,15]. However, there are scarce and conflicting data regarding the associations between renal function and the outcomes of ablation procedures. For instance, a single-center retrospective study reported that renal dysfunction significantly increases the risk of AF recurrence after radiofrequency (RF) or cryoablation therapy [16]. Moreover, the likelihood of AF recurrence during a mean follow-up period of 20 months was found to increase with worsening kidney function. Additionally, a retrospective multicenter analysis of AF patients treated with cryoablation found that mild to moderate reductions in renal function were associated with an increased risk of AF recurrence during a 2-year follow-up period [17]. In contrast, an evaluation of commercial claims and Medicare supplemental databases, which assessed 30-day safety and 1-year clinical outcomes in 21,091 patients who underwent a first AF ablation procedure, found that CKD was not independently associated with AF hospitalization, cardioversion, or repeat ablation [18].

The objective of this study was to evaluate the real-world effectiveness of PVI in patients with CKD by comparing the incidence of AF recurrence in CKD patients to those without CKD, utilizing eGFR data from the Israeli AF Ablation Prospective Registry.

## 2. Methods

The Israeli Catheter Ablation Registry (ICAR) is a prospective multicenter registry of all the patients undergoing AF ablation in 14 electrophysiology centers in Israel. The registry is a collaborative effort among the national communities of cardiac electrophysiologists and is managed by the Israeli Center for Cardiovascular Research (ICCR) based at Sheba Medical Center, Tel Hashomer, Israel. The registry was approved by the ethics committee of each participating institution, and all the patients provided written informed consent. Pulmonary vein anatomy was determined based on periprocedural imaging. Patient demographic characteristics, patient-specific procedural data, and outcomes were compared between patients with normal renal function and patients with CKD.

For this analysis, we included patients who underwent their first PVI between the years 2019–2021 and had available baseline serum creatinine data. While data were collected prospectively, this specific analysis was conducted retrospectively in 2025.

Data were collected prospectively, at the time of the index AF ablation hospital admission, by the local electrophysiologist and entered into a secure web-based electronic case report form, using Research Electronic Data CAPture (REDCap^®^) tools (https://projectredcap.org/software/ (accessed on 28 August 2025)) [19].

Baseline characteristics recorded included patient age, sex, type of AF, concomitant and previous antiarrhythmic drugs (AADs), comorbidities, calculated CHA_2_DS_2_-VASC score, left ventricular ejection fraction, left atrial (LA) size, and baseline creatinine, hemoglobin, and glucose levels. Procedural data recorded included the type of sedation, ablation strategy, type of energy used, procedure time, periprocedural and procedural imaging, fluoroscopy time, pulmonary vein anatomy, and acute complications.

Three CKD categories were defined according to varying levels of eGFR: Preserved or high GFR (eGFR > 90 mL/min/1.73 m^2^), mildly reduced eGFR (60–89 mL/min/1.73 m^2^), and moderately to severely reduced eGFR (<59 mL/min/1.73 m^2^). The GFR was calculated according to the 2021 CKD Epidemiology Collaboration (CKD-EPI) equation [20], using the laboratory parameters at the time of the index AF ablation hospital admission; no data were available regarding proteinuria.

Acute and chronic complications were recorded, including tamponade, thromboembolism, stroke, phrenic nerve paralysis, heart block, pericarditis, vascular complications requiring intervention or prolonged hospital stay, atrioesophageal fistulae, and death. Electrical cardioversions for recurrent AF, repeat ablation procedures, or antiarrhythmic therapy were documented. Follow-up was conducted through telephone interviews with patients and by reviewing subsequent clinic visits and hospital records in case of rehospitalization. A typical protocol for institutional follow-up included patient visits at 3 and 12 months post procedure, with an ECG and a 48 h ECG Holter monitor at each visit, or at any time for symptoms suggestive of arrhythmia recurrence. After ablation, all the patients were discharged on oral anticoagulation for at least 3 months. Oral anticoagulation and antiarrhythmic medications were discontinued at the discretion of the treating physician.

Ablation was performed under conscious sedation or general anesthesia according to local practice. The ablation procedure, based on the electrical isolation of the pulmonary veins (PVI), was performed as previously described, using the cryoballoon technique in the majority of the patients or point-by-point radiofrequency ablation guided by 3D electroanatomical mapping at the discretion of centers and operators [21,22,23,24]. Additional cavotricuspid isthmus radiofrequency ablation, in the event of documented right atrial flutter, was performed at operator discretion.

The primary effectiveness endpoint was the first recurrence of AF lasting for at least 30 s after a PVI procedure. Secondary endpoints were the repeat of any ablation and all-cause rehospitalization after PVI. The secondary safety endpoints were the clinical outcomes and procedural complications after AF ablation according to varying eGFR levels compared to the preserved eGFR population.

Statistical analysis: Patients’ characteristics were presented as *n* (%) for categorical variables and as the mean (SD) or median (IQR) for normally/non-normally distributed continuous variables. A chi-squared test for the trend was used for the comparison of categorical variables. Analysis of variance with one degree of freedom was performed for the comparison of normally distributed continuous variables and Kendall rank correlation for non-normal distributions. Uni- and multivariable Cox proportional hazard models were used to assess the impact of the eGFR level on the outcome of recurrent AF in 1 year. Covariates for the multivariable model were selected based on univariable analysis (*p* < 0.05). No imputation of missing data was conducted on covariate analysis, since the selected variables had <10% missing values.

All the tests were conducted at a two-sided overall 5% significance level (α = 0.05). All the analyses were performed using R software (R Development Core Team, version 4.0.0, Vienna, Austria).

## 3. Results

Between January 2019 and December 2021, a total of 1002 AF patients underwent PVI at the participating centers. Baseline creatinine was available for 929 patients, which comprised the study cohort. Of these, 226 (24%) had preserved eGFR (>90 mL/min/1.73 m^2^), 511 (55%) had mild eGFR reduction, and 192 (21%) had moderately to severely reduced eGFR (<60 mL/min/1.73 m^2^), qualifying for the definition of CKD.

The patients’ baseline characteristics are presented in Table 1. Patients with moderately to severely reduced eGFR were generally older and more likely to be female. There were no clinically meaningful differences in the use of antiarrhythmic medications among the eGFR groups, either before or after PVI. The burden of comorbidities was significantly higher among patients with moderately to severely reduced eGFR compared to those with preserved renal function. These patients had greater prevalences of diabetes mellitus, dyslipidemia, hypertension, prior myocardial infarction, prior coronary artery bypass graft (CABG) surgery, severe left ventricular dysfunction, cerebrovascular disease (CVA/TIA), and a higher CH_2_DS_2_-VASC score. The median AF duration was 3 years in all the groups. Persistent AF was significantly more common in patients with a lower eGFR. No significant differences were observed in the left atrium’s size or volume among the three groups. The procedural characteristics are outlined in Table 2. The majority of the patients (85%) underwent cryoablation, 8% underwent RF ablation, and 7% underwent both cryo- and RF ablations. There were no significant differences in procedural characteristics among the study groups.

## 4. Study Outcomes

**Figure 1:**
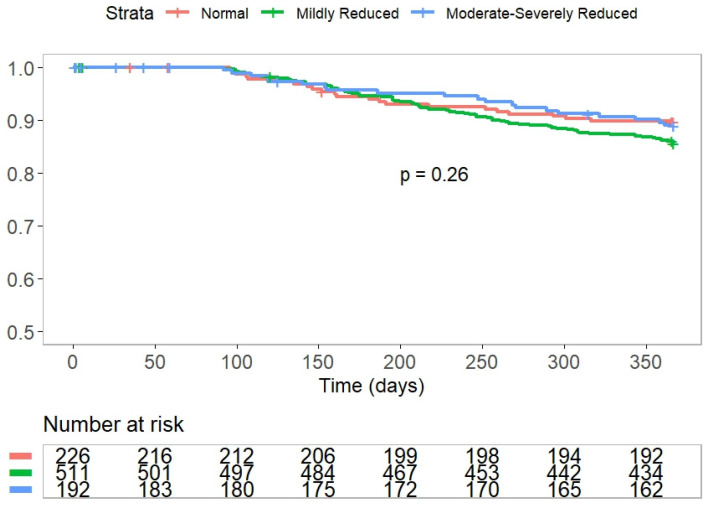
Atrial fibrillation and recurrence rate according to eGFR category.

The overall AF recurrence rate at 12 months post ablation was 33%. There were no significant differences in 12-month AF recurrence rates among the three study groups after a blanking period of 90 days (30%, 32%, and 40% in the patients with preserved eGFR, mild, and moderately to severely reduced eGFR, respectively (*p*-value for the trend = 0.1)) (Figure 1, Table 3). The lack of a significant association remained following a multivariable adjustment including relevant confounders, such as age, sex, heart failure, hypertension, AF subcategory, left atrial size, antiarrhythmic drug use, and AF ablation procedure type. Using preserved eGFR as a reference, demonstrated hazard ratios (HRs) for 12-month AF recurrence were 1.2 (95% CI, 0.75–2.2) and 0.7 (95% CI, 0.35–1.42) for patients with mild and moderately to severely reduced eGFR, respectively. Moreover, when eGFR was analyzed as a continuous variable, no statistically significant association was found between eGFR and the risk of AF recurrence (HR = 0.92) (95% CI, 0.84–1.02) per 10 mL/min/1.73 m^2^ decline. There were no significant differences in either the rate or the time to repeat AF ablation among the study groups. Overall, nine (1% of the) patients died during the 1-year follow-up period. The one-year mortality rate was higher in patients with mild and moderately to severely reduced eGFR compared with the preserved eGFR group (0%, 0.8%, and 2.7% in patients with preserved eGFR, mild, and moderately to severely reduced eGFR, accordingly (*p* for the trend = 0.02)). Six out of the nine patients (67%) died due to a non-cardiac cause (Table 3).

The one-year rehospitalization rate was higher in patients with moderately to severely reduced eGFR (19%, 24%, and 32% in patients with preserved eGFR, mild, and moderately to severely reduced eGFR, accordingly (*p* for the trend = 0.01) (Table 3)). Moreover, heart failure readmissions were significantly higher in patients with advanced CKD (5%, 4%, and 18% in patients with preserved eGFR, mild, and moderately to severely reduced eGFR, accordingly (*p* for the trend < 0.01) (Table 3)). Periprocedural complications were infrequent and did not vary significantly among the study groups (Table 4).

## 5. Discussion

This prospective study evaluated the associations between renal function, as assessed based on eGFR, and outcomes of PVI in patients with AF. Despite the higher burden of comorbidities in patients with a lower eGFR, our study found no significant difference in AF-recurrence-free survival at 12 months post ablation, as demonstrated by the Kaplan–Meier curve (Figure 1, *p* = 0.26). However, patients with a lower eGFR had higher one-year rates of HF rehospitalization and all-cause mortality, without a corresponding increase in other cardiac-related hospitalizations or cardiac mortality.

CKD is a common comorbidity in AF and is associated with elevated risks for cardiovascular complications, including ischemic stroke and cardiovascular mortality [8,9,10,11]. These associations are not completely understood and appear to be multifactorial and likely bidirectional. Patients with reduced renal function tend to be older and have more comorbidities, including coronary artery disease, HF, and diabetes. Moreover, the use of AADs and direct oral anticoagulants is often limited in this group [7,15,25] partly because the increased thromboembolic risk associated with CKD is not adequately captured by the CHA_2_DS_2_-VA score [15].

PVI has become a standard of care therapy for AF [13,15], yet patients with advanced CKD are frequently excluded from clinical trials—particularly those evaluating anticoagulation for stroke prevention and rhythm control therapy [7,26,27,28], resulting in limited evidence to guide the clinical management of AF in the CKD population.

In the present study, PVI demonstrated a favorable safety profile across all the eGFR groups, with low periprocedural complication rates. Antiarrhythmic use during follow-up was similar among the groups and did not significantly affect outcomes. The predominance of cryoballoon ablation in our cohort reflects current clinical practice at the participating centers, favoring this technique due to its procedural efficiency and ease of use. Radiofrequency ablation was reserved for selected cases based on operator preference. Previous studies have demonstrated comparable safety and efficacy between cryoballoon and radiofrequency ablations [29,30], suggesting that the predominance of cryoballoon ablation in our study is unlikely to have significantly influenced the overall outcomes.

Higher non-cardiac rehospitalization and overall mortality rates in patients with moderately to severely reduced eGFR may be attributed to the natural history of HF and CKD. Indeed, these patients were generally older, with lower LVEFs and higher rates of IHD (Table 1). Importantly, only three of the nine deaths were cardiac related (Table 3). The absence of excessive procedural complications (Table 4) supports the hypothesis that PVI was not the cause of increased mortality and HF readmission rates.

Previous large retrospective US studies have reported higher complication rates in CKD patients: In one study, the CKD group had a higher overall acute complication rate, which was predominantly driven by bleeding complications [29]. In a second study [18], patients with CKD had a fourfold increased risk of hospitalization due to HF. However, evolving ablation techniques and technologies limit the comparability of these findings to our contemporary prospectively followed cohort, which showed lower complication rates. Given the substantial burden of AF in CKD and the limited efficacy and tolerability of pharmacological therapies—compounded by drug interactions with agents like potassium binders and diuretics [31,32]—as well as the challenges faced when antiarrhythmic drugs are necessary, catheter ablation may provide a valuable alternative.

Nevertheless, the present study has several limitations that warrant consideration. First, as this is a registry, there is a possibility of case underreporting, both in terms of cases not submitted to the registry and AF recurrence episodes that may have gone undetected, given the absence of a standardized monitoring protocol. Patients with severely reduced eGFR (<30 mL/min/1.73 m^2^) and end-stage kidney disease (ESKD; eGFR < 15) were underrepresented in the cohort (0.5% and 0.6% of the study population, respectively) compared to the other groups. This may be due to selection bias for the procedure, as discussed above. Consequently, the results may not be generalizable to this population, which often presents with more complex pathophysiology and comorbidity profiles. Further dedicated studies are needed to clarify the safety and efficacy of AF ablation in these high-risk groups. Second, the study results and conclusions are mostly relevant to patients who underwent PVI using cryoablation, as only 8% of the study population underwent RF ablation. Third, the analysis relied solely on eGFR as a marker of renal function, as albuminuria, proteinuria, and other indicators of renal damage were not consistently available. This limits our ability to fully characterize CKD stages. Additionally, the registry did not systematically collect data on the precise etiology of atrial fibrillation, which limits our ability to explore how specific underlying mechanisms of AF may interact with renal function to influence outcomes as well. Finally, the ICAR is a comprehensive national survey of the Israeli population, necessitating cautious extrapolation of our findings to other countries. Randomized controlled trials are imperative to further explore the efficacy of AF ablation in CKD patients, particularly those with severe CKD and ESKD.

Our findings are consistent with recent large-scale trials, such as CABANA [18] and EAST-AFNET [33], which demonstrated both the relative safety and clinical benefits of catheter ablation in early rhythm control of AF management. However, detailed subgroup analyses based on CKD status remain unpublished in these trials. Our study contributes valuable real-world evidence on the safety and effectiveness of PVI, specifically in patients across the spectrum of renal dysfunction. Future randomized studies focused on the CKD population are warranted to confirm and expend upon these findings.

## 6. Conclusions

PVI is a safe and effective procedure that should be considered for CKD patients with atrial fibrillation who are deemed suitable for the intervention, even in the presence of declined eGFR values. Future studies are still needed to evaluate the safety and effectiveness of the procedure in individuals with a severely reduced eGFR or end-stage kidney disease.

## Figures and Tables

**Table 1:** Baseline characteristics according to eGFR.

Baseline Characteristics		* eGFR Level		*p*-Value
	Normal	Mildly Reduced	Moderately to Severely Reduced	
n (929)	226 (24%)	511 (55%)	192 (21%)	
Age, years (median)	61	66	70	<0.001
Gender, male, n (%)	162 (71)	316 (62)	103 (53)	<0.001
CHA_2_DS_2_-VASC, median [IQR]	2 [1, 3]	2 [2, 3]	3 [2, 4]	<0.01
Dyslipidemia, n (%)	105 (46)	251 (49)	123 (64)	<0.001
Hypertension, n (%)	110 (49)	324 (63)	157 (82)	<0.001
Diabetes mellitus, n (%)	55 (24)	107 (21)	73 (38)	<0.001
AF duration (median)	3.0 (1.0–4.8)	3.0 (1.0–6.0)	3.0 (1.0–5.0)	0.54
eGFR median	102 (96–112)	74 (68–82)	51 (44–55)	<0.001
Prior MI, n (%)	10 (4)	44 (9)	33 (17)	<0.001
Prior CABG, n (%)	6 (2)	13 (2)	12 (6)	0.04
Prior CVA/TIA, n (%)	19 (8)	40 (8)	26 (13)	0.05
LV dysfunction, n (%)				
Mild	20 (10)	44 (10)	19 (11)	0.01
Moderate	8 (4)	28 (6)	18 (10)	0.01
Severe	5 (3)	19 (4)	16 (9)	0.01
LA size, mm (median)	42	42	43	0.1
LA volume, cc (median)	58	62	65	0.6
AF classification, n (%)				
Paroxysmal	165 (73)	337 (66)	106 (56)	<0.001
Persistent	52 (23)	165 (32)	73 (38)	<0.001
Long-standing persistent	8 (4)	7 (1)	10 (5)	0.2
Permanent	0 (0)	1 (0.2)	1 (0.5)	0.2
Anticoagulant and antiarrhythmic therapies, n (%)				
AAD drugs (baseline)	134 (59)	349 (68)	133 (69)	0.03
AAD drugs (after ablation)	150 (66)	361 (70)	139 (72)	0.3
Prior anticoagulant therapy	177 (78)	453 (89)	183 (96)	<0.001
Apixaban	110 (62)	279 (62)	109 (60)	0.2
Dabigatran	18 (10)	55 (12)	19 (10)	0.2
Rivaroxaban	46 (26)	104 (23)	42 (23)	0.3
Warfarin	3 (2)	15 (3)	12 (7)	0.2

* eGFR categories: Preserved (>90 mL/min/1.73 m^2^), mildly reduced (60–89 mL/min/1.73 m^2^), and moderately to severely reduced (<60 mL/min/1.73 m^2^). CKD = chronic kidney disease; MI = myocardial infarction; CABG = coronary artery bypass graft; CVA = cerebrovascular accident; TIA = transient ischemic attack; LV = left ventricle; LA = left atrium; AAD = antiarrhythmic drug.

**Table 2:** Pulmonary vein isolation procedure information.

		* eGFR Level		
	Preserved	Mildly Reduced	Moderately to Severely Reduced	*p*-Value
n (929)	226	511	192	
Procedure performed				0.88
Cryoablation, n (%)	194 (86)	428 (84)	162 (84)	
RF Ablation, n (%)	15 (7)	45 (9)	17 (9)	
Cryo- and RF ablation, n (%)	17 (7)	38 (7)	13 (7)	
Procedure duration, minutes (median)	90	85	80	0.3
Fluoroscopy time, minutes (median)	23	21	21	0.7
General anesthesia, n (%)	120 (53)	269 (53)	112 (59)	0.2
Cardiac CT, n (%)	104 (46)	233 (46)	78 (42)	0.5
TEE (before/during), n (%)	95 (42)	199 (39)	93 (49)	0.06
Use of ICE, n (%)	59 (26)	114 (22)	39 (20)	0.3

* eGFR categories: Preserved (>90 mL/min/1.73 m^2^), mildly reduced (60–89 mL/min/1.73 m^2^), and moderately to severely reduced (<60 mL/min/1.73 m^2^). RF ablation = radiofrequency ablation; Cardiac CT = cardiac computed tomography; TEE = transesophageal echocardiography; ICE = intracardiac echocardiography.

**Table 3:** Study outcomes according to eGFR.

		* eGFR Level		
	Preserved	Mildly Reduced	Moderately to Severely Reduced	*p*-Value
n (929)	226	511	192	
AF recurrence **, n (%)	63 (30)	150 (32)	69 (40)	0.1
Repeat ablation for recurrent AF, n (%)	13 (6)	47 (10)	22 (13)	0.09
Time to first re-AF, months (median)	4.8	5.2	2.4	0.4
Time to first re-ablation, months (median)	9.8	7.9	10	0.9
1-year all-cause mortality, n (%)	0 (0)	4 (0.8)	5 (2.7)	0.02
1-year cardiac mortality, n (%)	0 (0)	1 (0.1)	2 (1)	N/A
Rehospitalizations (12 months), n (%)	39 (19)	111 (24)	55 (32)	0.01
Cause of rehospitalizations, n (%)				
Heart failure	2 (5)	4 (4)	10 (18)	<0.01
Cardiac	26 (72)	85 (78)	36 (69)	0.4
Scheduled	12 (30)	37 (34)	22 (40)	0.6

* eGFR categories: Preserved (>90 mL/min/1.73 m^2^), mildly reduced (60–89 mL/min/1.73 m^2^), and moderately to severely reduced (<60 mL/min/1.73 m^2^). ** After 90-day blanking period. AF = atrial fibrillation; re-AF = recurrent atrial fibrillation; re-ablation = repeated ablation; HF = heart failure; N/A= numbers were too small to calculate *p*-value.

**Table 4:** Periprocedural complications.

		* eGFR Level		
	Preserved	Mildly Reduced	Moderately to Severely Reduced	*p*-Value
n (929)	226	511	192	
Pericardial effusion, n (%)	1 (0.4)	0 (0)	0 (0)	0.2
Tamponade, n (%)	1 (0.4)	0 (0)	0 (0)	0.2
Cardiac arrest, n (%)	0 (0)	1 (0.2)	0 (0)	0.6
Thromboembolic events, n (%)	0 (0)	0 (0)	1 (0.5)	0.1
Neurological events, n (%)	1 (0.4)	3 (0.6)	3 (0.6)	0.33

* eGFR categories: Preserved (>90 mL/min/1.73 m^2^), mildly reduced (60–89 mL/min/1.73 m^2^), and moderately to severely reduced (<60 mL/min/1.73 m^2^).

## Data Availability

The data that support the findings of this study are available from the Israeli Center for Cardiovascular Research (ICCR) on reasonable request, although they will be subjected to data privacy rules and requirements of the Institutional Review Board. The original contributions presented in this study are included in the article/Supplementary Materials. Further inquiries can be directed to the corresponding author.

## Supplementary Materials

The following supporting information can be downloaded at: https://www.mdpi.com/article/10.3390/jcm14176227/s1, Table S1: Distribution of missing values; Table S2: Unadjusted and adjusted cox regression models for AF recurrence.
