# Supplementary material for: Outcomes of Atrial Fibrillation Ablation in Patients with Chronic Kidney Disease
Source: J Clin Med. 2025 Sep 3;14(17):6227. doi: 10.3390/jcm14176227 (PMC12429049; doi:10.3390/jcm14176227)
Supplement: Supplementary file 1 [file jcm-14-06227-s001.zip › jcm-3779898 - SI.pdf]

Supplementary Data

Table S1: Distribution of missing values

Variable	Proportion missing %
Age	0.1
Gender male	0
CHA_2DS_2-VASC	0.1
Dyslipidemia	0
Hypertension	0.2
Diabetes mellitus	0.1
AF duration	9.7
eGFR	
Prior MI	0.1
Prior CABG	0.1
Prior CVA/TIA	0
LV dysfunction, n (%)	9.2
LA size	9.2
AF classification	9.2
AAD drugs	9.2
Prior anticoagulant therapy	0.2

Table S2: Unadjusted and Adjusted Cox Regression Models for AF Recurrence.

Variable	Unadjusted (HR, 95% CI)	P Value	Adjusted model (HR, 95% CI) *	P Value
**GFR**				
Normal GFR	Reference		Reference	
Mildly Reduced GFR	1.41 (0.87- 2.27)	0.15	1.29 (0.75-2.24)	0.35
Moderate-Severely Reduced GFR	1.06 (0.58-1.95)	0.83	0.69 (0.34-1.42)	0.32
Age (per 10 years)	1.22 (1.1-1.48)	0.039	1.01 (0.98-1.03)	0.42
Sex (male)	0.44 (0.30-0.65)	<0.001	0.58 (0.37-0.89)	0.014
CHA2DS2-VASC (per 1-point increase)	0.99 (0.86-1.13)	0.88	-	
Persistent AF	1.43 (0.97-2.11)	0.06	1.16 (0.73-1.82)	0.51
AF duration (per year)	1.01 (0.97-1.06)	0.52	-	
Atrial flutter	1.30 (0.83-2.05)	0.25	-	
Congestive HF	1.03 (0.59-1.77)	0.92	1.44 (0.77-2.67)	0.24
Hypertension	0.94 (0.63-1.40)	0.75	0.75 (0.48-1.18)	0.21
Dyslipidemia	0.95 (0.65-1.39)	0.78	-	
Stroke/TIA	0.93 (0.47-1.85)	0.84	-	
Thromboembolic events	1.31 (0.48-3.56)	0.59	-	
Bundle branch block	0.86 (0.43-1.71)	0.66	-	
Beta blockers	0.91 (0.61-1.36)	0.65	-	
Antiarrhythmic drugs	1.50 (0.97-2.32)	0.07	2.76 (1.78-4.29)	0.001
**LA size**				
Normal	Reference		Reference	
Mild enlargement	1.42 (0.84-2.39)	0.19	1.26 (0.73-2.19)	0.39
Moderate enlargement	1.38 (0.72-2.64)	0.33	1.37 (0.707-2.68)	0.34
Severe enlargement	2.96 (1.31-6.69)	0.01	2.63 (1.13-6.12)	0.024
LV dysfunction	0.63 (0.35-1.16)	0.13	-	
**Sedation type**				
Minimal sedation	Reference		-	
Moderate sedation	0.75 (0.40-1.39)	0.35	-	
Deep sedation	0.79 (0.31-1.98)	0.61	-	
General anesthesia	0.95 (0.56-1.62)	0.86	-	
Use of any imaging	1.89 (0.88-4.08)	0.11	-	-
**Procedure Performed**				
Cryo	0.98 (0.47-2.02	0.96	0.79 (0.38-1.67)	0.54
RF	1.56 (0.66-3.69)	0.30	1.23 (0.64-2.34)	0.52

Abbreviations: HR (hazard ratio), CI (confidence interval), TIA (transient ischemic attack); AF (atrial fibrillation); LA (left atrium); HF (heart failure). *Variables with $P < 0.05$ in univariable analyses were included in the model, and established risk factors were forced into the model regardless of statistical significance.
